# Eczema Herpeticum Secondary to Atopic Dermatitis: A Case Study

**DOI:** 10.51894/001c.144603

**Published:** 2025-09-30

**Authors:** Neysa Miller

**Affiliations:** 1 College of Osteopathic Medicine Michigan State University

## 101

### INTRODUCTION

Atopic Dermatitis is a chronic inflammatory skin condition thought to be caused by a genetic defect in the filaggrin protein. The condition is characterized by pruritic, erythematous, and scaly skin lesions most often on the flexural surfaces. The skin defects caused by atopic dermatitis weakens the skin barrier increasing the risk of skin infections in patients. Eczema Herpeticum, also known as Kaposi’s varicelliform eruption, is one of these possible infections. It is caused by herpes simplex virus (HSV) infection of the skin and presents as clusters of vesicles possibly with systemic symptoms. It can even progress to a disseminated infection, which can cause serious life-threatening complications. HSV-1 exposure is not known to cause Eczema Herpeticum without an underlying disruption of the skin barrier, therefore, it is important to understand the relationship between these two conditions.

While in Iquitos, Peru, we had the opportunity to care for a 6-year-old female with a classic presentation of Eczema Herpticum secondary to atopic dermatitis.

### CASE REVIEW

The 6 year old female presented with the complaint of a painful rash on right hip for the past 4 days. The patient’s caretaker reported that she has had a similar breakout on her cheek in the past that cleared up on its own over time. We found diffuse xerosis, diffuse hypopigmentation on her distal legs, dry plaques on her elbows, allergic shiners, and Dennie-Morgan folds all lead to the diagnosis of atopic dermatitis. The erythematous vesicular rash on the right hip and focal erythematous lesion in the suprapubic region on the right lead us to the final diagnosis of eczema herpticum secondary to atopic dermatitis. The patient was prescribed Acyclovir 400 mg daily for 7 days and educated about the importance of keeping her skin moisturized.

### DISCUSSION/CONCLUSION

Eczema Herpeticum is a potentially life-threatening disease and is known to be unexpectedly rare. The prevalence has been known to be more common in areas with high rates of sexually transmitted disease. The goal is to raise more awareness and hopefully bring more preventive tactics to light.

